# Protocol for evaluation of perioperative risk in patients aged over 75 years: Aged Patient Perioperative Longitudinal Evaluation–Multidisciplinary Trial (APPLE-MDT study)

**DOI:** 10.1186/s12877-020-01956-3

**Published:** 2021-01-06

**Authors:** Yanhong Zhang, Lina Ma, Tianlong Wang, Wei Xiao, Shibao Lu, Chao Kong, Chaodong Wang, Xiaoying Li, Yun Li, Chunlin Yin, Suying Yan, Ying Li, Kun Yang, Piu Chan, APPLE-MDT Study Working Group

**Affiliations:** 1 Medical Administration Division, Xuanwu Hospital of Capital Medical University, Beijing, 100053 China; 2 China National Clinical Research Center for Geriatric Disorders, Beijing, 100053 China; 3 Department of Geriatrics, Xuanwu Hospital of Capital Medical University, Beijing, 100053 China; 4 Department of Anesthesiology, Xuanwu Hospital of Capital Medical University, Beijing, 100053 China; 5 Department of Orthopaedics, Xuanwu Hospital of Capital Medical University, Beijing, 100053 China; 6 Department of Cardiology, Xuanwu Hospital of Capital Medical University, Beijing, 100053 China; 7 Department of Pharmacy, Xuanwu Hospital of Capital Medical University, Beijing, 100053 China; 8 Department of Nutrition, Xuanwu Hospital of Capital Medical University, Beijing, 100053 China; 9 Department of Evidence-based Medicine, Xuanwu Hospital of Capital Medical University, Beijing, 100053 China; 10 Department of Neurology and Neurobiology, Xuanwu Hospital of Capital Medical University, Beijing, 100053 China; 11 Department of Neurology, Xuanwu Hospital of Capital Medical University, Beijing, 100053 China

**Keywords:** Advanced-age patients, Perioperative assessment, Age-related surgical risk factors, Multidisciplinary evaluation

## Abstract

**Background:**

With the extended life expectancy of the Chinese population and improvements in surgery and anesthesia techniques, the number of aged patients undergoing surgery has been increasing annually. However, safety, effectiveness, and quality of life of aged patients undergoing surgery are facing major challenges. In order to standardize the perioperative assessment and procedures, we have developed a perioperative evaluation and auxiliary decision-making system named “Aged Patient Perioperative Longitudinal Evaluation–Multidisciplinary Trial (APPLE-MDT)”.

**Methods:**

We will conduct a perioperative risk evaluation and targeted intervention, with follow-ups at 1, 3, and 6 months after surgery. The primary objective of the study is to evaluate the effectiveness of the “Aged Patient Perioperative Longitudinal Evaluation-Multiple Disciplinary Trial Path” (hereinafter referred to as the APPLE-MDT path) in surgical decision-making for aged patients (≥75 years) undergoing elective surgery under non-local anesthesia in the operating room. The secondary objectives of the study are to evaluate the postoperative outcome and health economics of the APPLE-MDT path applied to the surgical decision-making of aged patients (≥75 years) undergoing elective surgery under non-local anesthesia and to optimize intervention strategies for aged patients undergoing surgery to reduce the occurrence of postoperative complications and improve the quality of life after surgery.

**Discussion:**

It is necessary to formulate a reliable, effective, and concise evaluation tool, which can effectively predict the perioperative complications and mortality of aged patients, support targeted intervention strategies, and allow for a more comprehensive risk and benefit analysis, thereby forming an effective senile perioperative surgery management path. It is expected that the implementation of this protocol can reduce the occurrence of postoperative complications, improve the postoperative quality of life, shorten hospital stay, reduce hospitalization expenses, reduce social burden, and allow the elderly to have a good quality of life after surgery.

**Trial registration:**

ChiCTR, ChiCTR1800020363, Registered 15 December 2018.

**Supplementary Information:**

The online version contains supplementary material available at 10.1186/s12877-020-01956-3.

## Background

The proportion of the world population aged ≥60 years will increase from 11.0% in 2010 to 21.8% in 2050 while the portion of aged ≥80 years will increase from 1.5 to 4.3% [1]. Aged patients, especially those over 75 years, more likely suffer from problems such as frailty, geriatric syndromes, comorbidities, polypharmacy, decline of organ function, and deteriorating brain health. As a result, the operability of aged patients decreases. Moreover, the risk of postoperative complications is significantly higher than that of other patients, and the rehabilitation process after surgery is slow. As a result, the safety, effectiveness and quality of life of aged patients undergoing surgery are facing major challenges.

In recent years, with an increase of aged patients undergoing surgery, perioperative management has become the focus of scientific research. Surgeons and anesthetists have gradually realized the importance of multidisciplinary comprehensive evaluation of aged patients undergoing surgery. The comprehensive geriatric assessment (CGA) is defined as a multi-dimensional and interdisciplinary process, which focuses on determining the medical, psychological, and functional capabilities of the frail elderly to formulate a coordinated and comprehensive treatment and long-term follow-up plan [2]. Since elderly patients often have multiple pathologies, preoperative assessment frequently calls for a multidisciplinary approach applying the concepts of CGA to guide the patient through the perioperative period [3]. Currently studies have found that the components of CGA can be used as predictors of postoperative complications among geriatric patients undergoing surgery. CGA results showed that patients with adverse outcomes were associated with functional dependency and poor nutrition [4, 5]. The cumulative number of impairments in the CGA domains was significantly associated with adverse outcomes, in-hospital events, and prolonged hospital stays [4]. Aged patients undergoing intervention after evaluation result in shorter hospital stay and reduced mortality [6]. It is suggested that multidisciplinary perioperative management plays an important role in elderly patients undergoing surgery.

However, perioperative management of the elderly is a complicated process, which needs more evidence to support best practices. The quantity of clinical trials available is really miserable. Some studies have shown that the complexity of CGA causes certain limitations in application [7]. Although CGA as a measure of preoperative evaluation are well recognized, there is no gold standard assessment that is universally used in the clinical and research setting.

This study aims to develop a perioperative multidisciplinary evaluation system for aged patients over 75 years undergoing surgery, which could identify and stratify risks, formulate preoperative, intraoperative and postoperative intervention strategies. We, therefore, construct a standardized “Longitudinal Perioperative Evaluation of Aged People (APPLE)” System for perioperative multidisciplinary evaluation and auxiliary decision-making for aged patients, and apply the APPLE-MDT model to guide the perioperative management of aged patients undergoing elective surgery under non-local anesthesia in the operating room, so as to optimize the treatment plan for aged patients and enhance the ability of aged patients to resist external stress with the goal of improving the outcome of such patients.

## Methods and design

### Study aim

This study will aim to develop the APPLE-MDT system and conduct multidisciplinary perioperative evaluation and intervention on the study group using the APPLE-MDT auxiliary system.

The primary goal of the perioperative assessment is to develop the perioperative multidisciplinary evaluation path (hereafter referred to as the APPLE-MDT path) in the surgical decision-making and intervention strategies for aged patients (≥75 years) undergoing elective surgery under non-local anesthesia in the operating room.

The secondary objective is to evaluate the effectiveness of the APPLE-MDT path with the postoperative outcome and health economics after applied to the aged patients (≥75 years) undergoing elective surgery, so as to reduce the occurrence of postoperative complications and improve the quality of postoperative life.

### Study design and setting

**Fig. 1:**
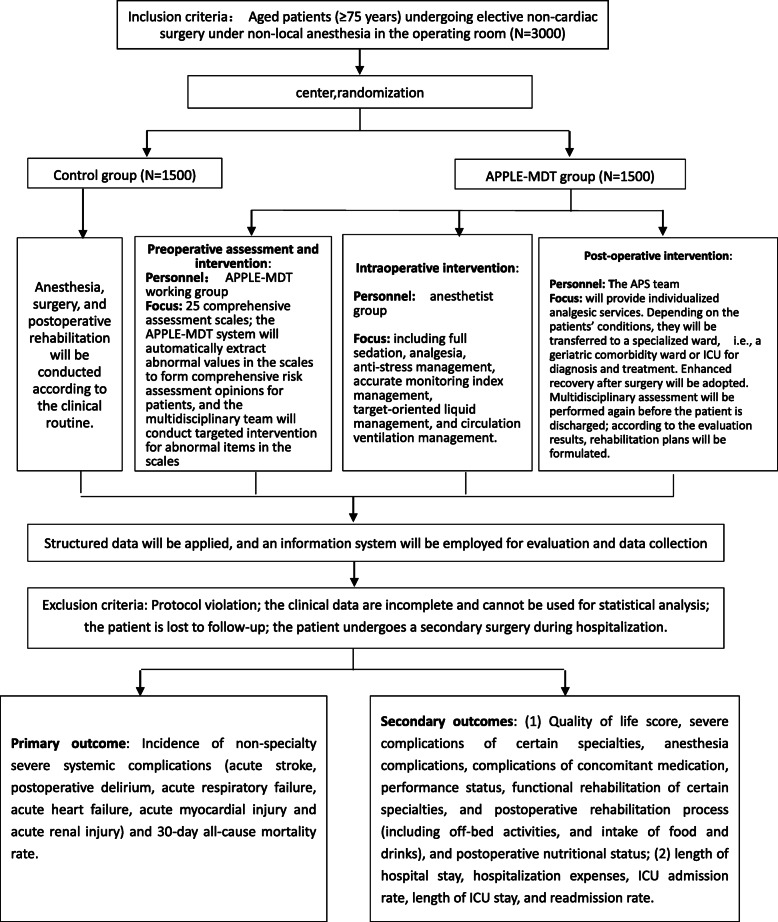
Study flow chart

This study is a single-center, parallel, randomized, controlled study. This study will be carried out in Xuanwu Hospital, Capital Medical University, which is a tertiary teaching hospital in China. The control group will undergo anesthesia and surgery according to an established clinical routine. The APPLE-MDT information system will be used for the APPLE-MDT group to assist in perioperative clinical decision-making by identifying risks and providing suggestions for mitigating interventions to be carried out. Preoperative evaluation will be completed within 48 h after the patient is admitted to hospital. Follow up assessments will be conducted at 1, 3, and 6 months after surgery. The multidisciplinary team will include specialists in surgery, anesthesiology, geriatrics, neurology, cardiology, pharmacy, nutrition, nursing, and rehabilitation. The detailed study flow chart is shown in Fig. 1.

The study started in March 2019. The recruitment period will be 30 months, with a follow-up of 6 months. The results of the study will be expected in mid-2022.

### Randomization

Patients over 75 years scheduled for non-cardiac surgery under non-local anesthesia, who meet pre-specified eligibility criteria, will be enrolled and randomized into the control group or the APPLE-MDT group by computer software. The randomization scheme will be blinded by the complete randomization scheme blinding method. Neither the subjects nor the follow-up personnel will know the grouping.

### Intervention scheme

Two groups will be planned for this study.

Control group: Participants enrolled will receive anesthesia, surgery, and postoperative rehabilitation according to an established clinical routine.

**Fig. 2:**
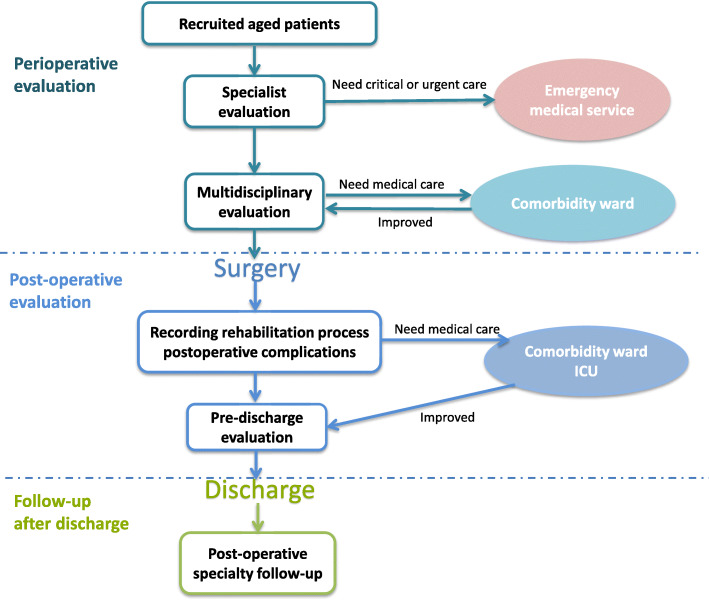
Aged Patient Perioperative Longitudinal Evaluation–Multidisciplinary Clinical Research (APPLE-MDT) clinical path

APPLE-MDT group (Fig. 2) will include:

1. Preoperative assessment (APPLE-MDT path)**:** A comprehensive multidisciplinary evaluation on 15 categories and 25 scales will be developed according to the recommendations of guidelines and consensus as well as consultation with the specialists [8–19] (Table 1). The APPLE-MDT system will automatically extract abnormal values on the relevant scales, identify likely perioperative risks, and recommend the comprehensive management opinions for patients. The multidisciplinary team will finally decide and conduct targeted intervention according to the APPLE-MDT system.
2. Intraoperative intervention: Following consideration of perioperative risks, the anesthetist group will implement individualized anesthesia schemes for each patient including full sedation, analgesia, anti-stress management, accurate monitoring indicator management, objectives-oriented liquid management, and circulation ventilation management, as appropriate.
3. Post-operative intervention: The acute pain service (APS) group will provide individualized analgesic service. Depending on the patients’ condition, the patients will be transferred to a specialized ward, geriatric comorbidity ward, or an ICU for diagnosis and treatment. Enhanced recovery after surgery will be carried out, and the rehabilitation process and postoperative variables of the patients will be recorded. Multidisciplinary evaluation will be conducted again before the patients are discharged; according to the evaluation results, rehabilitation plans (including the combined medication plan, nutritional state health guidance, functional state guidance plan, fall prevention plan, and rehabilitation precautions given by specialties, as required) will be formulated.
4. Follow-up: All subjects enrolled will undergo examination of postoperative complications, specialist follow-ups, and follow-ups of long-term quality of life (ADL, IADL) at 1, 3, and 6 months after surgery, respectively. In addition, an assessment of frailty (the Fried frailty phenotype scale) will be conducted at 6 months after surgery. The follow-up variables will be recorded. The follow-up assessments will be conducted during the patients’ hospital visits. Relevant web pages and mobile phone applications will be developed as remedial measures in case a follow-up cannot be conducted on site.

**Table 1:** Multidisciplinary Comprehensive Geriatric Assessment Scales

SYSTEM	SCALE	EVALUATION PERIOD		
		pre-operative evaluation	post-operative evaluation	follow-up at 6 months
Nutrition	Mini-Nutritional Assessment(MNA)	×	×	×
Functional Status	Activities of Daily Living Scale (ADL) Instrumental Activities of Daily Living Scale (IADL)	×	×	×
Frailty	Fried Frailty Phenotype Frailty Screening Questionnaire (FSQ)	×	×	×
Risk of Fall	Morse Fall Risk Assessment Scale	×	×	
Pain	Visual Analogue Scale (VAS)	×	×	
Respiratory System Risk Assessment	Arozullah Postoperative Respiratory Failure Predictive Score Scale Preoperative STOP-BANG Screening Diagnosis of Obstructive Respiratory Sleep Syndrome Predictive index of lung function for airway complications and mortality risk after operation	×		
Cardiac Risk Assessment	Cardiac evaluation in accordance with ACC/AHA Algorithm for non-cardiac surgery	×		
Cerebrovascular Risk Assessment	Modified Framingham Stroke Risk Score Essen Stroke Risk Score	×		
Liver Function and Renal Function	Medical history and laboratory test	×		
Blood Glucose	Laboratory test	×		
Cognitive Function	Mini-mental state examination (MMSE) Montreal Cognitive Assessment (MoCA) Risk Factors for Postoperative Delirium	×	×	
Anxiety and Depression	Self-rating Anxiety Scale (SAS) Self-rating Depression Scale (SDS)	×	×	
Thrombosis and Hemorrhage	Caprini Thrombosis Risk Factor Assessment Risk Assessment of Hemorrhagic Complications	×		
Polypharmacy	detailed medication history Appropriate perioperative adjustments	×	×	
Anesthesia-related Assessment	ASA Airway-related Assessment Intraspinal -related Assessment	×		

### Study outcomes

Primary outcomes: Incidence of non-specialty severe systemic complications (acute stroke, postoperative delirium, acute respiratory failure, acute heart failure, acute myocardial injury and acute renal injury) and all-cause mortality over the first 30 days.

Secondary outcomes: (1) Quality of life score, severe complications of certain specialties, anesthesia complications, complications of concomitant medication, performance status, functional rehabilitation of certain specialties, and postoperative rehabilitation process (including off-bed activities, intake of food and drinks, etc.), and postoperative nutritional status; (2) Length of hospital stay, hospitalization expenses, ICU admission rate, length of ICU stay, and readmission rate.

### Inclusion criteria

Patients are eligible for the study if they and their families agree with this protocol and sign an informed consent form. The patients must cooperate with the evaluation, have no contraindications to surgery, be over 75 years, not be undergoing local anesthesia, and not be undergoing elective cardiac surgery.

### Exclusion criteria

Patients are not eligible for the study if they are aged below 75 years; undergoing emergency or day surgery; undergoing surgery under local anesthesia; have an urgent condition that needs to be managed before the surgery, are unable to cooperate with the evaluation; they or their families refuse their participation in the study.

If a patient enrolled in the study requires a secondary surgery during hospitalization, the treatment will be carried out according to the diagnosis and established relevant routine; however, the subject will be excluded. If a subject misses any follow-up appointments or necessary clinical data cannot be obtained during the follow-up, the subject will be regarded as lost to follow-up.

### Sample size calculation

According to the literatures, the incidence of perioperative complications in the elderly is 20–28% [20–23]. It is expected that the APPLE-MDT group will reduce the incidence of complications by 20% compared with the control group. The APPLE-MDT group and the control group are matched by 1:1, assuming a power of 90% and a two-sided significance level (alpha) of 0.05, the sample size is demonstrated in Figure S1. It is estimated that the incidence of perioperative complications of the elderly in China is 26%, the sample size is 2776. Considering the loss of follow-up rate is 10%, 3054 participants will be randomized at a ratio of 1:1 into two groups. To establish a cohort, it is assumed that the number of clinical end points in one year is 10–20 times than the variables included in the model (20 variables). In this study, the incidence of end-point events was about 26%, considering the loss of follow-up rate is 20%, so 1847 cases are calculated. Therefore, 3054 patients recruited can meet the design requirements.

### Data collection, validation, and management

During the study, clinical monitors will regularly check the extracted data to ensure consistency with the contents of electronic medical records. Investigators in the clinical study will receive unified training to ensure standardization of data entry. The investigator should record the contents in the forms truthfully, carefully, and in detail according to the requirements of the study protocol to ensure the accuracy and reliability of the contents in the study medical records. All observations and results of laboratory examination will be verified to ensure the reliability of data and of all conclusions derived from this data. All data in this study will be collected automatically by the purpose-developed system. A patent will be applied for the system design scheme. The scores and formulas will be calculated based on the patients’ original data.

### Statistical analysis

Data will be presented as mean ± SD or mean rank or number and percentage. The difference in the characteristics between the two groups will be evaluated by chi-squared test for categorical variable, independent t-test for continuous variables, and Kruskal–Wallis comparisons for the abnormally distributed continuous variables, with adjustment for potential confounders by multivariable regression analysis. A Cox proportional hazards model was used to evaluate the effect of covariates on all-cause mortality. We considered *p* < 0.05 (two-tailed) statistically significant. Statistical analyses were performed with SPSS (Chicago, IL, USA, version 11.0).

## Discussion

Elderly patients undergo surgical interventions 4 times more often than the rest of the population [24]. The aging process determines a reduction of physiological reserve, which means that elderly person may become more vulnerable to stressors [25]. The prevalence of comorbidities and frailty is high. This aging process and clinical complexity especially creates a specific status that can modify the response to operations. This fact should prompt us to seek accurate clinical study that is specific to this age-group, so that there are reduced postoperative complications and mortalities for aged patients undergoing surgery.

When it comes to perioperative management of elderly patients, preoperative evaluation that can stratify risks and assist decision-making is very important.

The assessment of the surgical risk is currently performed by different instruments. Physiological and Operative Severity Score for the Enumeration of Mortality and Morbidity (POSSUM) [26], Surgical Risk Score (SRS) [27] that includes the National Confidential Enquiry into Patient Outcome of Death (NCEPOD) [28], American Society of Anesthesiologists (ASA) [29], and British United Provident Association (BUPA) are the more validated ones [30]. These scales are applied at any age, so they don’t take into account the characteristics of the elderly, such as frailty, functional declines.

CGA ensures that clinical and functional problems are identified, quantified, and managed appropriately by involving a multidisciplinary team [31]. There is literature demonstrates CGA can predict a higher risk of adverse outcomes independent of established surgical risk assessment tools [23]. The American College of Surgeons (ACS) recognizes the necessity for quality improvement in the surgical care of geriatric patients with the recent publication of guidelines for preoperative assessment in this population, including frailty assessment, and multiple components of the CGA (e.g., depression screening, nutritional status, cognitive ability, and functional status) [11]. Recently, significant improvements have been established in the perioperative management of hip fracture in aged patients. The effective collaboration between geriatrics and traumatology with the implementation of the orthogeriatric units have improved the outcomes [32]. Meta-analysis of orthopedic treatment after hip fracture has shown that the mortality rate and length of hospital stay following the surgery are relatively low [33]. However, preoperative evaluation of geriatric patients has not been standardized based on large-scale clinical evidence validated. Therefore, Perioperative management for aged patients still suffers from empirical, subjective in most institutions [31].

The purpose of our research is not to “identify” the risks of surgery itself. The objective of this study is to identify relevant medical problems and functional conditions, stratify the perioperative risks, predict potential perioperative problems, formulate optimal intervention plans. Therefore we choose these essential domains referred in guidelines and literatures, which are reliable, validated and brief to determine risk for morbidity and mortality in older patients, thereby forming an effective geriatric perioperative management path.

Traditionally, comprehensive geriatric preoperative assessment is time-consuming. We have developed “APPLE-MDT” information system which can efficiently and objectively collect evaluation data. This study verify the effectiveness of the “APPLE-MDT” perioperative management path, thus providing evidence for the perioperative management of aged patients. As some studies have shown that the risk of postoperative complications and death is not significantly related to the type of surgery [3], we will include patients undergoing different types of surgery in the study. As a result, this study may have a higher generalizability than those of studies performed in patients undergoing a single intervention.

At present, there is a lack of standard path and perioperative management of aged patients in China. We put forward a comprehensive and holistic study protocol that aims at evaluating perioperative risks of aged patients undergoing surgery in China, and further develops standardized strategies. It is expected that the implementation of this protocol can reduce the occurrence of postoperative complications, improve the postoperative quality of life, shorten hospital stay, reduce hospitalization expenses, reduce social burden, and allow the elderly to have a good quality of life after surgery.

## Supplementary Information

**Additional file 1: Figure S1.** Sample size calculation.

## Data Availability

Not applicable.

## Abbreviations

APS: Acute pain service
APPLE-MDT: Aged Patient Perioperative Longitudinal Evaluation-Multiple Disciplinary Trial
CGA: Comprehensive geriatric assessment
POSSUM: Physiological and Operative Severity Score for the Enumeration of Mortality and Morbidity
SRS: Surgical Risk Score
NCEPOD: National Confidential Enquiry into Patient Outcome of Death
ASA: American Society of Anesthesiologists
BUPA: British United Provident Association
ACS: the American College of Surgeons
