# Supplementary material for: Protocol for evaluation of perioperative risk in patients aged over 75 years: Aged Patient Perioperative Longitudinal Evaluation–Multidisciplinary Trial (APPLE-MDT study)
Source: BMC Geriatr. 2021 Jan 6;21:14. doi: 10.1186/s12877-020-01956-3 (PMC7788705; doi:10.1186/s12877-020-01956-3)
Supplement: Supplementary file 1 — Additional file 1: Figure S1. Sample size calculation. [file 12877_2020_1956_MOESM1_ESM.docx]

**Figure S1: Sample size calculation**
